# Leukemia Inhibitory Factor: A Potential Biomarker and Therapeutic Target in Pancreatic Cancer

**DOI:** 10.1007/s00005-021-00605-w

**Published:** 2021-02-25

**Authors:** Ewa Wrona, Piotr Potemski, Francesco Sclafani, Maciej Borowiec

**Affiliations:** 1 Department of Clinical and Laboratory Genetics, Medical University of Lodz, Lodz, Poland; 2 Department of Chemotherapy, Medical University of Lodz, Copernicus Memorial Hospital, Lodz, Poland; 3 Gastrointestinal Unit, Department of Medical Oncology, Institut Jules Bordet – Université Libre de Bruxelles (ULB), Brussels, Belgium

**Keywords:** Pancreatic ductal adenocarcinoma, Leukemia inhibitory factor, Biomarker, Therapeutic target

## Abstract

Pancreatic ductal adenocarcinoma (PDAC) is a highly aggressive, treatment-resistant cancer. Five-year survival rate is about 9%, one of the lowest among all solid tumors. Such a poor outcome is partly due to the limited knowledge of tumor biology, and the resulting lack of effective treatment options and robust predictive biomarkers. The leukemia inhibitory factor (LIF) has recently emerged as a potential biomarker and therapeutic target for PDAC. Accumulating evidence has suggested that LIF plays a role in supporting cancer evolution as a regulator of cell differentiation, renewal and survival. Interestingly, it can be detected in the serum of PDAC patients at higher concentrations than healthy individuals, this supporting its potential value as diagnostic biomarker. Furthermore, preliminary data indicate that testing for LIF serum concentration or tissue expression may help with treatment response monitoring and prognostication. Finally, studies in PDAC mouse models have also shown that LIF may be a valuable therapeutic target, and first-in-human clinical trial is currently ongoing. This article aims to review the available data on the role of LIF in PDAC promotion, and to discuss the evidence supporting its potential role as a biomarker and target of effective anti-cancer therapy in this setting.

## Introduction

Pancreatic cancer is one of the most lethal malignancies. Histopathologic type of ductal adenocarcinoma (PDAC) constitutes more than 90% of all pancreatic cancer cases. For all cancers combined, the 5-year relative survival rate between 2009 and 2015 in the USA was 67% (Siegel et al. 2020). For PDAC this was only 9% (Siegel et al. 2020). Almost as many deaths (*n* = 47,050) as new cases (*n* = 57,600) have been estimated to occur in 2020 in the USA, making PDAC the fourth leading cause of cancer-related death (Siegel et al. 2020). Based on the data collected between 2008 and 2017, the mortality trend for this disease raised by 0.3 AAPC (average annual percent change) which is in stark contrast with other cancer types such as lung or colorectal cancer (CRC), where substantial improvement in treatment has been made, and mortality actually dropped by − 3.3 and − 2.1 AAPC, respectively (Siegel et al. 2020). The highest age-standardised incidence rates of PDAC are recorded in Europe and Northern America, with mortality rates fourfold higher in high Human Development Index countries (Bray et al. 2018).

High mortality rate in PDAC patients can be partially explained by many factors including the biological tumor aggressiveness, the strongly immunosuppressive tumor microenvironment (TME) and the inherent chemoresistance. There is still limited knowledge of the mechanisms underlying PDAC growth, resistance to treatment and progression, this ultimately resulting in the lack of robust predictive and prognostic biomarkers and scarce availability of effective treatment options. CA 19-9 is the only biomarker approved for PDAC by Food and Drug Administration in 2002. Since then, no improvement has been made in this area. As far as systemic treatment is concerned, only marginal progress has been made especially if compared with other tumor types and, with the only exception of erlotinib and olaparib, the therapeutic armamentarium for PDAC is orphan of targeted treatments that could substantially improve survival.

**Fig. 1:**
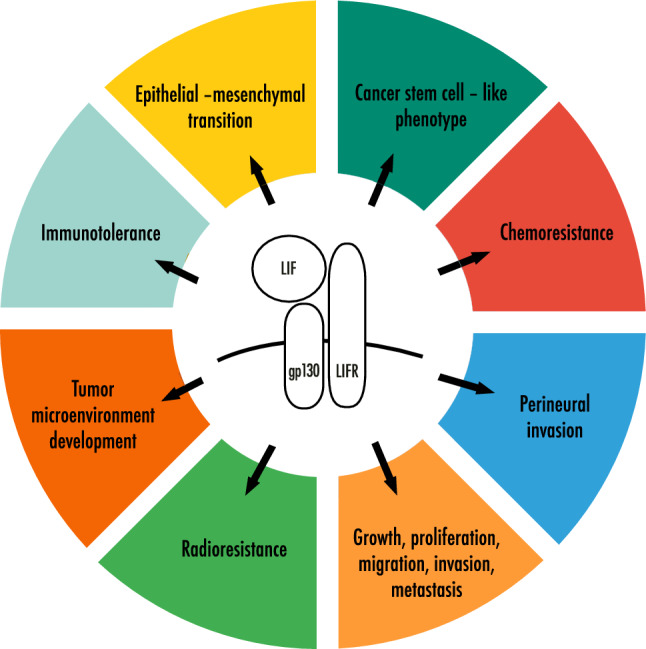
Cancer-promoting cellular functions of LIF

The leukemia inhibitory factor (LIF) is a cytokine involved in a number of physiological processes including regulation of cell differentiation, renewal and survival. Further to the results of preliminary studies supporting its role also in the mechanisms of PDAC promotion (Fig. 1), a substantial interest has recently emerged for the investigation of LIF in this disease setting. In particular, based mostly on preclinical studies, there are growing expectations for its potential as biomarker and therapeutic target.

In this article, we provide an overview of LIF involvement in key cancer-promoting processes. Furthermore, possible clinical applications of this cytokine in the setting of PDAC are discussed.

## The LIF Molecule and its Physiological Role

LIF was named after its ability to trigger final differentiation and prevent continuous growth of myeloid leukemic cells (Gearing et al. 1987). It is a member of the pro-inflammatory IL-6 family, together with IL-6, IL-11, IL-27, ciliary neurotrophic factor, cardiotropin 1, cardiotropin-like cytokine and oncostatin M, the latter being structurally the most similar to LIF (Wang et al. 2019). LIF gene is located on 22nd chromosome and has three splicing variants: LIF-D, LIF-M and LIF-T. Both LIF-D and LIF-M, the secreted variants, have a 22-amino-acids long secretion signal sequence which is lacking in LIF-T, the intracellular transcription variant (Haines et al. 2000).

LIF is a widely expressed cytokine with diverse patterns of glycosylation. As a paracrine and autocrine factor, LIF binds to LIFR and the gp130 dimeric receptor on the targeted cell surface. Although gp130 forms numerous heterodimers, with IL-6R for instance for IL-6 signal transduction, LIFR exclusively binds to LIF and the numbers of LIFR molecules on the cell surface increase during LIF exposure (Gulluoglu et al. 2017). The intracellular domain of the LIFR/gp130 dimer is capable of activating JAK kinase upon LIF-binding, which is the main, while not exclusive, pathway for LIF signal transduction. JAK kinase activation results in phosphorylation of the STAT3 transcription factor. In turn, phosphorylated STAT3 forms dimers and translocates into the nucleus, where it activates targeted gene transcription. LIF-JAK/STAT3 is a recognised signalling pathway for PDAC, CRC, ovarian cancer and non-small lung cell cancer (NSCLC). Of note, LIF was shown to activate alternative signalling pathways e.g. YAP/TEAD and AKT/mTOR (Table 1) (Li et al. 2014b; Mclean et al. 2019; Xia et al. 2014).

**Table 1:** Summary of the main functions of LIF in cancer promotion

Cellular function	Mechanism	Molecular pathway	Tumor types
Epidermal-mesenchymal transition	↓ EMT genes expression, ↓ adhesion	Hippo, Wnt, JAK/STAT3 pathway	PDAC (Shi et al. 2019)
Perineural invasion	Paracrine stimulation of Schwann cells	JAK/STAT3 pathway	PDAC (Bressy et al. 2018)
Immunotolerance	↑Monocyte M2 and Treg ↓T cytotoxic	↑CXCL9 expression (in case of M2 monocytes)	Glioblastoma, ovarian cancer (Pascual-García et al. 2019)
Cancer stem cell-like phenotype	Differentiation with hold	YAP/TEAD pathway	PDAC (Wang et al. 2019), glioblastoma (Bao et al. 2006)
Tumor microenvironment development	Autocrine CAFs activation	JAK/STAT3 pathway	PDAC (Albrengues et al. 2014; Bressy et al. 2018)
Chemoresistance	Stem cell-like phenotype; EMT induction, p53 downregulation and degradation → 5FU resistance; increased drug transporter expression	YAP/TEAD, Hippo, Wnt, JAK/STAT3 pathway, ID1, MDM2, p53, ABCG2↑ expression	PDAC (Shi et al. 2019), Colorectal cancer (Yu et al. 2014), chordoma (Gulluoglu et al. 2017)
Radioresistance	↓DNA damage response	mTOR/p70S6K	Nasopharyngeal cancer (Liu et al. 2013)
Growth, proliferation, progression, invasion, metastasis	Transcription factors activation, ECM remodelling	JAK/STAT3, AKT/mTOR pathway	PDAC (Albrengues et al. 2014; Mclean et al. 2019; Shi et al. 2019; Wysoczynski et al. 2007)

PDAC pancreatic ductal adenocarcinoma, LIF leukemia inhibitory factor, EMT epidermal-mesenchymal transition, Treg regulatory T leukocyte, CAF cancer associated fibroblast, 5FU 5-fluorouracil, ECM extracellular matrix

LIF is known to control differentiation, survival and renewal processes that are regulated in time and tissue-dependent manner (Nicola and Babon 2015). It has been extensively studied as an inflammation modulator. Early studies in embryology found that LIF can create an immunosuppressive microenvironment to enable implantation and to protect the embryo throughout pregnancy (Piccinni et al. 2001). LIF regulates the development and proliferation of regulatory T cells (Tregs) by stimulating the expression of Foxp3, inhibiting RORγt in CD4^+^T cells and suppressing the development of IL-6-induced Th17 lineages (Gao et al. 2009). Studies in transplantation medicine and autoimmune diseases also confirmed LIF as an immunosuppressing factor (Metcalfe et al. 2005). In addition, LIF inhibits adipocyte lipoprotein lipase activity, suppresses stem cell differentiation, maintains adrenocorticotropic hormone secretion in the pituitary gland, plays a role in lung vasculature and alveolus development, influences muscle organ morphogenesis, induces embryonic stem cell self-renewal, neuron development and remodelling, wound healing, and protects from ischemic injury (Bauer and Patterson 2006; Baumann et al. 1987; Haines et al. 2000; Metcalf 2003; Moreau et al. 1987; Nicola and Babon 2015; Niwa et al. 1998; Silver and Hunter 2010).

## LIF Roles in Cancer

### Tumor Microenvironment Development

TME is one of the hallmarks of cancer (Hanahan and Weinberg 2011). Research on the relationship between the tumor and its TME, have shown the development of the TME occurs during the early stages of tumorigenesis. TME contributes to the growth, progression, invasion, metastatic potential and drug resistance of cancer (Hanahan and Weinberg 2011; Ligorio et al. 2019; Murakami et al. 2019). It is composed of numerous cell populations, including cancer-associated fibroblasts (CAFs), accounting for the majority of cells, tumor-associated macrophages (TAMs), Tregs, B lymphocytes, dendritic cells, endothelial cells and pericytes (Hanahan and Weinberg 2011). Activated CAFs in PDAC models were shown to be the ones triggering and maintaining TME development by inducing extracellular matrix (ECM) remodelling and recruitment of immunosuppressive cells into the tumor neighbourhood (Fu et al. 2018; Gao et al. 2010; Hwang et al. 2019; Ligorio et al. 2019; Pascual-García et al. 2019).

Although pro-invasive CAFs were previously thought to be activated and maintained by transforming growth factor (TGF)-β direct stimulation, this contention was questioned by the results of a recent study showing mediation of LIF that directly binds on the surface of CAFs and induces their activation via the JAK/STAT3 pathway (Albrengues et al. 2014). Analysis of the TME in PDAC tissue samples demonstrated diverse levels of LIF expression depending on cell type. Low levels of expression were observed in epithelial cells (LIF and cytokeratin-19 co-staining detected in only 6.8% of cells), while high expression was detected in mast cells, macrophages and CAFs, where mean counts of cells expressing LIF were 21.8, 34.4 and 47.5%, respectively (Bressy et al. 2018). Interestingly, among all cell types with marked LIF expression, CAFs were the only ones capable of secreting LIF into the PDAC TME (Bressy et al. 2018). In addition, proteomic studies identified LIF as the key paracrine factor responsible for interactions between TME and pancreatic cancer cells. Secreted by CAFs in the microenvironment, LIF was shown to bind on the surface of pancreatic cancer cells, induce STAT3 phosphorylation, and ultimately stimulate PDAC growth and progression (Albrengues et al. 2014; Shi et al. 2019). LIF was also proven to be a strong chemo-attractant for immunosuppressive cell recruitment into the TME which will be described further in the review (Pascual-García et al. 2019). Altogether, these studies indicate that LIF acts as: (1) an autocrine factor secreted by CAFs and able to self-stimulate regardless of TGF-β; (2) a paracrine stimulator of ECM remodelling, recruitment of TME components and pancreatic cancer cells growth (Albrengues et al. 2014; Bressy et al. 2018; Fu et al. 2018; Ligorio et al. 2019; Sada et al. 2016).

### Tumor Growth, Progression, Invasiveness and Metastasis

ECM remodelling, epithelial-mesenchymal transition (EMT) and JAK/STAT3 pathway-dependent genes transcription in pancreatic cancer cells are three primary mechanisms responsible for the maintenance of PDAC growth, invasion and metastatization. The ECM is a complex network of non-cellular components, mostly collagen fibres, that is responsible for cell-to-cell interactions, adhesion and proliferation. ECM remodelling occurs through increased deposition of polarised collagen bundles, tissue stiffening and metalloproteinase overexpression. This results in a number of events such as cell invasiveness along aligned collagen fibres, a pro-metastatic environment phenotype, insensitivity to growth inhibitors, and self-maintained growth and angiogenesis (Giussani et al. 2019). Its contribution to tumor evolution is universal across multiple cancer types. Pro-invasive ECM remodelling was shown to be mediated by LIF paracrine stimulation. Indeed, LIF concentration correlates with the density of polarised collagen fibres in Sirius Red staining of xenograft model samples, as well as cancer cells invasiveness and poor clinical outcome (Albrengues et al. 2014). In particular, stimulation of the ECM remodelling was mediated by the JAK/STAT3 pathway activation in fibroblasts, while JAK1/2 inhibition (ruxolitinib) or LIF depleting therapy (anti-LIF antibody) had an inhibitory effect (Albrengues et al. 2014).

As a paracrine factor, LIF binds to the heterodimer of LIFR and gp130 on the surface of pancreatic cancer cells, activates the JAK/STAT3 pathway and ultimately induces transcription of genes which are known to regulate cell cycle, apoptosis, angiogenesis and invasion (Dauer et al. 2005; Shi et al. 2019). Such an activation results in self-sustained pancreatic cancer cell growth, proliferation and migration. STAT3 activation was proven to be exclusively dependent on the presence of LIF in in vivo PDAC models where its phosphorylation was effectively halted upon *LIFR* knockdown or LIF ligand immunodepletion (Albrengues et al. 2014; Mclean et al. 2019; Shi et al. 2019). Of note, LIF targeted therapy resulted in a statistically significant decrease in tumor growth as compared to untreated controls (Mclean et al. 2019). Additionally, the effect of anti-LIF blocking antibodies could not be counteracted by upstream TGF-β stimulation (Albrengues et al. 2014).

Finally, mesenchymal transition is known to entail a loss of cell adhesion molecules e.g. E-cadherin, thus contributing to the acquisition by cancer cells of a migratory phenotype (Thomas et al. 2020). In PDAC mouse models, EMT was reported to be promoted by peripheral infusion of LIF, while it was suppressed in the presence of anti-LIF antibodies (Shi et al. 2019).

### Perineural Invasion

Perineural invasion (PNI) is a poor prognostic factor in PDAC, being associated with higher local recurrence rate, shorter survival and increased neuropathic pain (Bapat et al. 2011; Chen et al. 2010). PNI was detected in up to 75% of stage I tumor samples, suggesting it is a process that takes place early during PDAC progression (Hirai et al. 2002; Pour et al. 2003). The mechanisms underlying PNI in PDAC have not been fully elucidated. According to some studies, the sonic Hedgehog pathway may play a role in stimulating PNI in this disease setting (Li et al. 2014a).

Interestingly, LIF secretion by CAFs in the TME was shown to activate the JAK/STAT3 pathway in adjacent Schwann cells and lead to PNI, thus unravelling another axis of PDAC promotion (Bressy et al. 2018). Paracrine LIF activity can increase the neuronal plasticity, migration and differentiation of Schwann cells, an effect that strongly depends on LIF concentration, contributes to the process of PDAC-associated neural remodelling, and is directly linked to PNI (Bressy et al. 2018). On a larger scale, it leads to neoneurogenesis and axonogenesis during early PDAC development (Bressy et al. 2018). A study suggests a correlation between serum circulating LIF level and intra-PDAC nerve density in mice models, with substantial decrease in nerve density achieved upon LIF-targeted therapy. A similar association between circulating LIF and intra-tumor nerve density was also observed in human PDAC samples (Bressy et al. 2018).

### Immunotolerance

Two of the most typical hallmarks of PDAC, especially if compared with other solid cancers, are the enrichment in dense stroma (which accounts for up to 70% of the tumor tissue area) and the poorly immunogenic microenvironment (Blando et al. 2019). The decreased immunologic response and inflammatory processes of the PDAC microenvironment are mainly secondary to the abundance of macrophages and Tregs which are attracted to the tumor neighbourhood (Blando et al. 2019). In this regard, LIF seems to be one of the major driving factors for the recruitment of immunosuppressive subpopulations of lymphocytes into the TME. While there are no studies on the immunosuppressive effect of LIF in PDAC, this contention is supported by pre-clinical evidence from another poorly immunogenic solid tumor, such as glioblastoma. Pascual-García et al. (2019) showed that LIF-dependent decrease in CXCL9 expression and CCL2 overexpression underlie recruitment of TAMs and Tregs and regulation of CD8^+^ T cell infiltration. In their study, anti-LIF antibody treatment led to tumor growth suppression and increased survival in mouse models through the reduction of pro-tumoral M2 macrophages and Tregs, as well as the increase of tumor-infiltrating CD8^+^ T cell and NK cells. Increased CXCL9 expression upon anti-LIF therapy was also associated with the recruitment of a CD8^+^PD-1^+^ T cell subpopulation into the TME. Interestingly, combined treatment with anti-LIF antibody and PD-1 blockade yielded better response compared with either treatment alone. Moreover, an immunological memory was observed which successfully prevented tumor re-inoculation in mice with complete responses (Pascual-García et al. 2019).

### Cancer Stem Cell Self-renewal

Maintaining a cancer stem cell-like phenotype leads to increased chemoresistance, invasion, infiltration and poor outcome (Penuelas et al. 2009). The ability to maintain a spherical shape or sustain expression of antigens such as CD133, CD24, CD44, nestin, CXCR4, EpCAM, ABCG2 or c-Met, are considered as hallmarks of cancer stem cells. In contrast to what has been reported for acute myeloid leukemia, in PDAC LIF paracrine activity induces cancer stem cell self-renewal and cell differentiation arrest (Gearing et al. 1987; Hoffman-Liebermann and Liebermann 1991; Penuelas et al. 2009; Shi et al. 2019; Wang et al. 2019). A study in KRAS mutated PDAC (which account for approximately 90% of all PDAC cases) identified LIF as a driving factor for sphericity maintenance and CD44 expression (Wang et al. 2019). Of all factors of the IL-6 family, only LIF was found to regulate stem cell self-renewal (Wang et al. 2019). This effect was reported to be mediated by Hippo suppression and later activation of the YAP/TEAD pathway. LIF silencing by shRNA or CRISPR/Cas9 led to a loss of the ability of human and mouse pancreatic cancer cell lines to grow as spheres, an effect which was reversible after exposure to culture medium with LIF (Wang et al. 2019). Of note, aforementioned study reported also blocked sphere-forming ability upon LIF knock out with later increase in the sensitivity to several chemotherapeutics, i.e. gemcitabine, 5-FU and cisplatin (Wang et al. 2019). Combination treatment with anti-LIF antibody (10 mg/kg twice a week) and gemcitabine (100 mg/kg once a week) was reported to result in complete tumor remissions in seven out of nine patient-xenograft mouse models (PDX), which was maintained after treatment withdrawal. In contrast, after an initial response, rapid progression was observed in gemcitabine-only treated PDX models, underlining the importance of LIF-depleting therapy in the combination treatment arm in limiting stem cell-like phenotype following with chemosensitivity (Wang et al. 2019).

### Chemo and Radioresistance

Various molecular mechanisms (including stem cell-like phenotype maintenance, EMT induction and overexpression of drug transporters) are known to contribute to treatment resistance in PDAC. As discussed already, maintaining cancer stem cell-like phenotype contributes largely to chemoresistance (Gulluoglu et al. 2017; Mclean et al. 2019; Shi et al. 2019; Wang et al. 2019). Additionally, EMT was reported to be markedly reduced, along with the inhibition of the downstream Hippo, Wnt and STAT3-signalling, upon combined anti-LIF antibody and gemcitabine treatment, but not upon gemcitabine monotherapy (Shi et al. 2019). Combination treatment also resulted in substantial increase in cell differentiation, apoptosis and prolonged survival compared to mice treated with gemcitabine alone (Shi et al. 2019).

LIF role in chemoresistance mechanisms was studied more extensively in other cancer types than PDAC. In colorectal cancer, LIF was shown to induce chemoresistance through a p53-dependent mechanism. In short, LIF downregulated p53 through STAT3 activation, resulting in ID1 induction (Yu et al. 2014). In turn, ID1 upregulated MDM2, a well-known negative regulator of p53, and triggered p53 proteasomal degradation by E3 ubiquitin ligase (Yu et al. 2014). 5-FU, a key drug in both PDAC and CRC treatment, induces p53-mediated apoptosis by caspase 3 cleavage in p53^+/+^ mice while this phenomenon is greatly reduced in p53^–/–^ mice or p53 wild type with peripheral LIF infusion. Finally, LIF can promote chemoresistance by enhancing the activity of drug transporters. A small study in chordoma cell lines U-CHI and MUG-Chor1 demonstrated that LIF stimulation can increase the expression of the drug transporter ABCG2 on the cell surface (Gulluoglu et al. 2017).

The association between LIF expression and radioresistance in PDAC has never been studied. Preclinical data from nasopharyngeal cancer, however, suggest that LIF can activate mTORC1/p70S6K signalling that in turn would inhibit DNA damage response and induce radioresistance (Liu et al. 2013). LIF blockade, as well as mTOR inhibition, resulted in increased sensitivity to gamma radiation (Liu et al. 2013).

### Cachexia

Cachexia is an established negative prognostic factor for cancer patients. LIF has long been known to have an impact on adipocyte lipolysis which is a cornerstone of cancer related cachexia. Only recently, however, studies in CRC mouse models have detailed the mechanism of body weight loss upon LIF stimulation which appears to be mediated by JAK/STAT3 pathway activation in adipocytes (Arora et al. 2018). It was reported that Lif^+/+^ mice had a 55–75% greater body weight loss, muscle loss, fat loss and splenomegaly when compared with a Lif^–/–^ knock out model while no impact on cardiac mass was observed (Kandarian et al. 2018). Unfortunately, no study has yet addressed the impact of LIF on cachexia in PDAC models.

## LIF as a Biomarker in Pancreatic Cancer

Based on the preliminary data, LIF seems to be a promising biomarker for PDAC. LIF concentration in human serum can be easily determined by ELISA testing. Available ELISA kits for research applications have a detection threshold that ranges widely from 0 up to 2000 pg/mL depending on the manufacturer (Elisa Q, HImmunoassay, H.L.I.F. 2014; Sheet 2017). Data on serum LIF levels in cancer have initially been made available by studies in mouse models. A study on circulating LIF in caerulein-induced PDAC models showed gradually increasing concentrations during progression from 5th week (*p* = 0.03) to 7th week (median LIF concentration level reaching 50 pg/mL) after caerulein exposure. In contrast, LIF serum concentrations were at the lowest levels of detection in a chronic pancreatitis model and in a control group (Shi et al. 2019). Accordingly, available studies in humans reveal large differences in median LIF concentrations between PDAC patients (200 pg/mL) and healthy controls (4 pg/mL) (Shi et al. 2019). Also, in line with preclinical findings, no significant differences appear to exist between samples from benign pancreatic disease and healthy controls (Bressy et al. 2018; Shi et al. 2019). Undoubtedly, these findings require confirmation in larger clinical study.

Predictive value of LIF serum levels in PDAC was also assessed in serial measurements during systemic treatment. Data from a small cohort of 14 patients, who were treated with preoperative chemotherapy and had serum samples available before and after treatment, revealed an association between variations of LIF levels and response. In addition, LIF was shown to be superior than CA 19-9 in predicting tumor response by RECIST1.1 (Shi et al. 2019).

Beyond the investigation of LIF as a potential circulating biomarker, expression of this cytokine (at both the mRNA and protein level) in PDAC tissue samples has also been assessed. In 77 PDAC patients LIF median tumor expression was up to sevenfold higher than in paired healthy pancreatic tissue samples (Shi et al. 2019). Of note, LIF protein and mRNA tissue expression increased gradually from healthy controls to patients with pancreatic cyst, chronic pancreatitis and finally PDAC (Bressy et al. 2018; Shi et al. 2019; Wang et al. 2019). Large differences in median LIF tissue concentration have also been reported between pancreatic cancer patients with well-differentiated tumors (456 pg/mg) and those with poorly-differentiated tumors at the time of diagnosis (1118 pg/mg) (Shi et al. 2019). A study of immunohistochemical staining, reported pancreatic intraepithelial neoplasias to express low levels of LIF, making it a potential candidate as early detection biomarker (Bressy et al. 2018).

Based on data from the TCGA database, *LIF* mRNA expression appeared to have a prognostic value. In a small (*n* = 33) cohort of early-stage (I or IIa) PDAC patients, high *LIF* mRNA expression was associated with shorter disease-free survival (DFS) when compared with low *LIF* mRNA expressing tumors (median DFS of 7 vs 19 months; *p* = 0.0067) (Shi et al. 2019). LIF tissue expression was also significantly lower in PDAC patients with overall survival exceeding 2 years compared with those with shorter survival (678 pg/mg vs 1150 pg/mg, respectively; *p* = 0.023) (Shi et al. 2019).

## Therapeutic Implications of LIF

Preliminary preclinical data on the role of LIF in cancer progression have prompted the design of a humanised anti-LIF monoclonal antibody (MSC-1). In orthotopic mouse models, this was found to be effective against various types of cancer including glioblastoma, NSCLC, ovarian cancer, CRC and PDAC (Seoane et al. 2017). In light of these interesting preliminary results, a phase I clinical trial of MSC-1 in high LIF-expressing cancer patients has been launched in the US, Canada and Spain (NCT03490669).

Since LIF engages exclusively with the LIFR/gp130 complex on PDAC cells and CAFs, LIFR appears to be an equally valuable target in disrupting the paracrine effect of LIF. LIFR inhibition has already been found to effectively block LIF-induced perineural invasion in pancreatic cancer cell lines and in mouse models (Bressy et al. 2018). Following these preclinical data, the National Institute of Health has supported a study analysing the safety and activity of the anti-LIFR antibody EC359 in combination with gemcitabine in human (Nair and Kumar 2019).

Unfortunately, two promising phase III clinical trials with inhibitors of JAK/STAT3 (i.e. the JAK1/JAK2 inhibitor ruxolitinib and the STAT3 inhibitor napabucasin), the leading effector pathway of LIF stimulation in cancer cells, were stopped due to futility (Boston Biomedical 2019; Hurwitz et al. 2018). Their failure suggests that blocking LIF’s primary downstream pathway may not exert similar effects as those observed by depleting LIF in preclinical studies. Also, alternative LIF downstream signalling pathways may exist and represent more relevant therapeutic targets than JAK/STAT3.

## Conclusion

LIF was extensively studied in embryology, immunology, transplantology and hematology over last 30 years. The renowned interest for this cytokine has recently been prompted by the discovery of analogies between LIF functions in aforementioned areas and cancer evolution. LIF is involved in a number of key processes underlying cancer growth and progression including immunotolerance, PNI, chemo and radioresistance, cancer stem cell-like phenotype maintenance, EMT, and TME development (Fig. 1, Table 1). Many aspects of its actual role in PDAC still require further investigation, since these are drawn mostly based on preclinical studies. Larger clinical studies are also needed to confirm the potential of this cytokine as biomarker for early detection, treatment response monitoring and prognostication. Finally, results of ongoing clinical trials will provide some insights regarding the value of LIF as therapeutic target in PDAC and other tumor types.
